# Physiotherapy Rehabilitation of Scapholunate Ligament Instability Following Ganglion Cyst in a Recreational Basketball Player: A Case Report

**DOI:** 10.7759/cureus.68257

**Published:** 2024-08-30

**Authors:** Bharat K Rathi, Chaitali S Vikhe, Swapnil U Ramteke, Pratik R Jaiswal

**Affiliations:** 1 Sports Physiotherapy, Ravi Nair Physiotherapy College, Datta Meghe Institute of Higher Education & Research, Wardha, IND

**Keywords:** sports physiotherapy, physical rehabilitation, sports related injuries, scapholunate ligament instability, ganglion cyst

## Abstract

Ganglion cysts are prevalent benign soft tissue tumors, commonly occurring on the dorsal wrist and often associated with underlying joint structures like the scapholunate ligament (SLL), a critical stabilizer of the wrist. SLL instability, frequently resulting from acute trauma or repetitive stress, can significantly impair wrist function, causing pain, reduced range of motion, and decreased grip strength. This case report details the conservative management of a 25-year-old recreational basketball player who presented with SLL instability and a dorsal ganglion cyst following two separate wrist injuries. Despite initial conservative management, the patient continued to experience persistent pain and functional limitations. Diagnostic imaging confirmed the presence of a ganglion cyst arising from the SLL, which necessitated a targeted physiotherapy regimen. The rehabilitation protocol focused on pain relief, wrist stability, muscle strengthening, and functional performance, employing phonophoresis, K-taping, laser therapy, and progressive strengthening exercises. Throughout treatment, the patient exhibited marked improvements in wrist range of motion, muscle strength, and pain reduction, ultimately returning to basketball activities without recurrence of symptoms. This case underscores the potential effectiveness of conservative physiotherapy in managing SLL instability with associated ganglion cysts, emphasizing the importance of a comprehensive, multifaceted approach to rehabilitation in restoring wrist function and enabling a return to sports.

## Introduction

Ganglion cysts are common wrist lesions in about 20% of patients, typically located in both volar and radial regions. They are the most prevalent benign soft tissue tumors of the hand, with 60-70% of these cysts occurring on the dorsal surface of the wrist [1-2]. These cysts are often adherent to an underlying joint capsule, ligament, or tendon sheath, or are attached via a pedicle [3]. The most plausible explanation for their formation is the migration of synovial cells into the joint capsule, where they secrete synovial fluid, leading to mucinous degeneration [4]. The scapholunate ligament (SLL), an essential primary stabilizer of the wrist, is the most frequently injured intercarpal ligament [5]. Scapholunate stability depends on a complex network of intrinsic and extrinsic ligaments, collectively known as the scapholunate complex [6]. Improved anatomical understanding of this complex allows for earlier and more accurate diagnosis of pathological lesions, facilitating more physiological treatment approaches [7]. Patients with SLL instability often present with pain, reduced range of motion, and loss of strength, significantly affecting their activities of daily living [8]. The recurrence rate of ganglion cysts after surgical excision ranges from 4% to 40%, with the rate of recurrence after revision surgery remaining unknown. Studies have not shown that nonsurgical treatments result in improved cyst resolution rates compared to observation alone [9-10]. Consequently, there is no definitive treatment for the pathology. To relieve pain, various treatments have been adopted, including supportive splinting, non-steroidal anti-inflammatory drugs, aspiration, and surgical procedures. While these therapies are commonly employed in clinical practice for ganglion cyst treatment, their effects are often unsatisfactory due to long treatment cycles and high recurrence rates [11].

This case report focuses on the conservative management of ganglion cysts, specifically through physiotherapy rehabilitation, in a recreational basketball player with scapholunate ligament instability.

## Case presentation

A 25-year-old recreational basketball player, previously in good health, started suffering diffuse pain in his left wrist following an incident at the gym in October 2023. While using a Smith machine, his neck ornament became entangled in his right hand, causing a sudden twist of his wrist. He endured mild pain for two months, managing it with analgesics and not seeking proper medical attention. Further, in December 2023, he suffered another injury while descending stairs. He missed a step, and in an attempt to prevent a fall, he grabbed a nearby bar, resulting in a twist of his left wrist. This incident exacerbated his pain, prompting him to seek medical care, where he was prescribed analgesics. Despite this, the pain persisted. After two months, he returned to the gym but struggled with significant pain and difficulty in gripping and lifting weights. Attempting self-management, he applied ice to the affected area but continued to experience discomfort. He subsequently sought help at a sports physiotherapy center. After a thorough consultation with an orthopedic specialist and undergoing the necessary diagnostic investigations, he was diagnosed with scapholunate ligament instability accompanied by a ganglion cyst in his left wrist. He was then referred to the sports physiotherapy outpatient department for specialized treatment.

Clinical findings

Upon initial presentation at the sports physiotherapy center, the patient reported pain in his left wrist, particularly exacerbated by gripping and lifting activities. Localized tenderness was present over the scapholunate region. A physical examination revealed a decreased range of motion of the left wrist which is mentioned in Table 1. Strength was also affected, as mentioned in Table 2. The swelling was absent but a palpable ganglion cyst was identified on the dorsal aspect of the left wrist over the same area. Radiological imaging included an MRI, which confirmed the diagnosis of scapholunate ligament instability and identified the presence of a ganglion cyst arising from the scapholunate ligament's dorsal band. Functionally, the patient had difficulty performing daily activities involving wrist movements, lifting objects, writing for prolonged periods, and playing basketball. He rated his pain intensity at 7/10 on the numerical pain rating scale during activity, reducing to 0/10 at rest. These clinical findings supported the diagnosis, which led to subsequent physiotherapy rehabilitation.

**Table 1:** Pre- and post-range of motion

Active movement	Painful range	Left		Right		Normal range
	Pre-intervention	Pre-intervention	Post-intervention	Pre-intervention	Post-intervention	
Flexion	Beyond 50°	0-50°	0-80°	0-80°	0-80°	0-80°
Extension	Beyond 60°	0-60°	0-70°	0-70°	0-70°	0-70°
Radial deviation	Beyond 15°	0-15°	0-20°	0-20°	0-20°	0-20°
Ulnar deviation	Beyond 20°	0-20°	0-30°	0-30°	0-30°	0-30°

**Table 2:** Pre- and post-manual muscle testing

Muscle group	Left		Right	
	Pre-intervention	Post-intervention	Pre-intervention	Post-intervention
Wrist flexors	4-/5	5/5	5/5	5/5
Wrist extensors	4-/5	5/5	5/5	5/5
Radial deviators	4-/5	5/5	5/5	5/5
Ulnar deviators	4-/5	5/5	5/5	5/5

Radiological investigations

**Figure 1:**
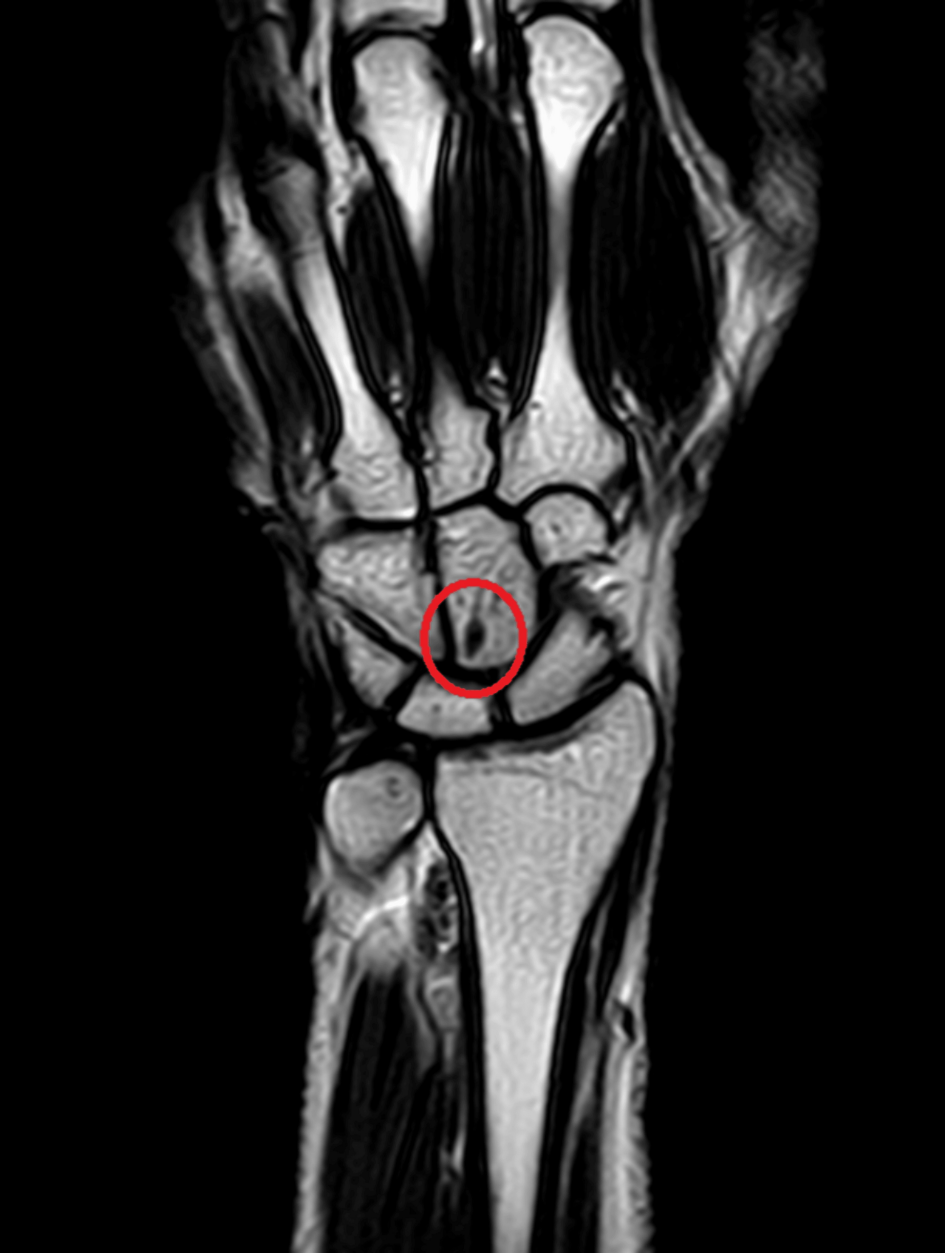
A tiny ganglion cyst was seen arising from a dorsal band of the scapholunate ligament of the left wrist as shown in the coronal section of the T1 weighted sequence

**Figure 2:**
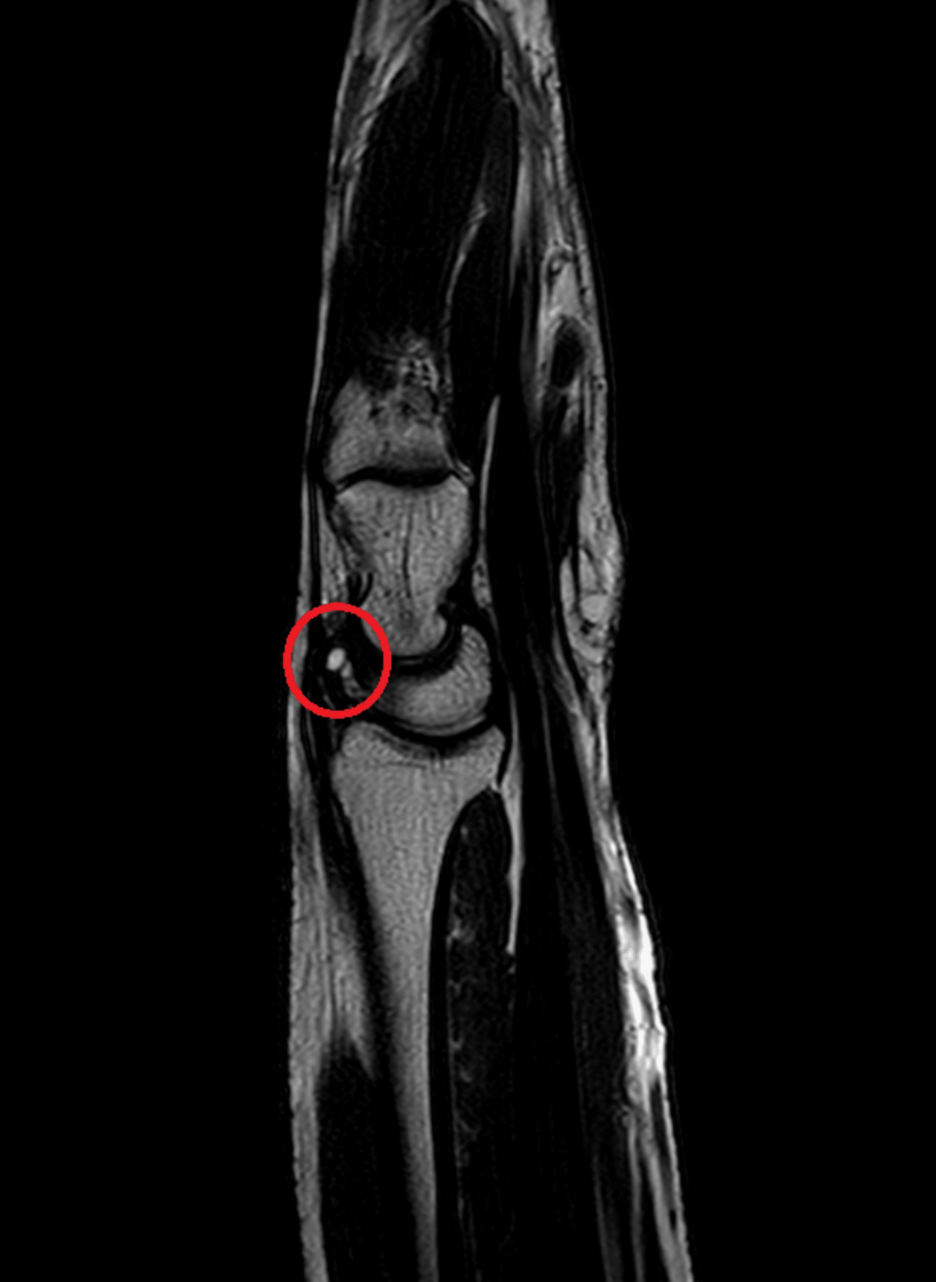
A tiny fluid signal intensity cystic lesion measuring 4 mm was seen arising from a dorsal band of scapholunate ligament of the left wrist along the dorsal aspect, appearing hyperintense as seen in the sagittal section of the T1 weighted sequence

Magnetic resonance imaging investigations have been depicted in Figure 1 and Figure 2.

Physiotherapy intervention

**Figure 3:**
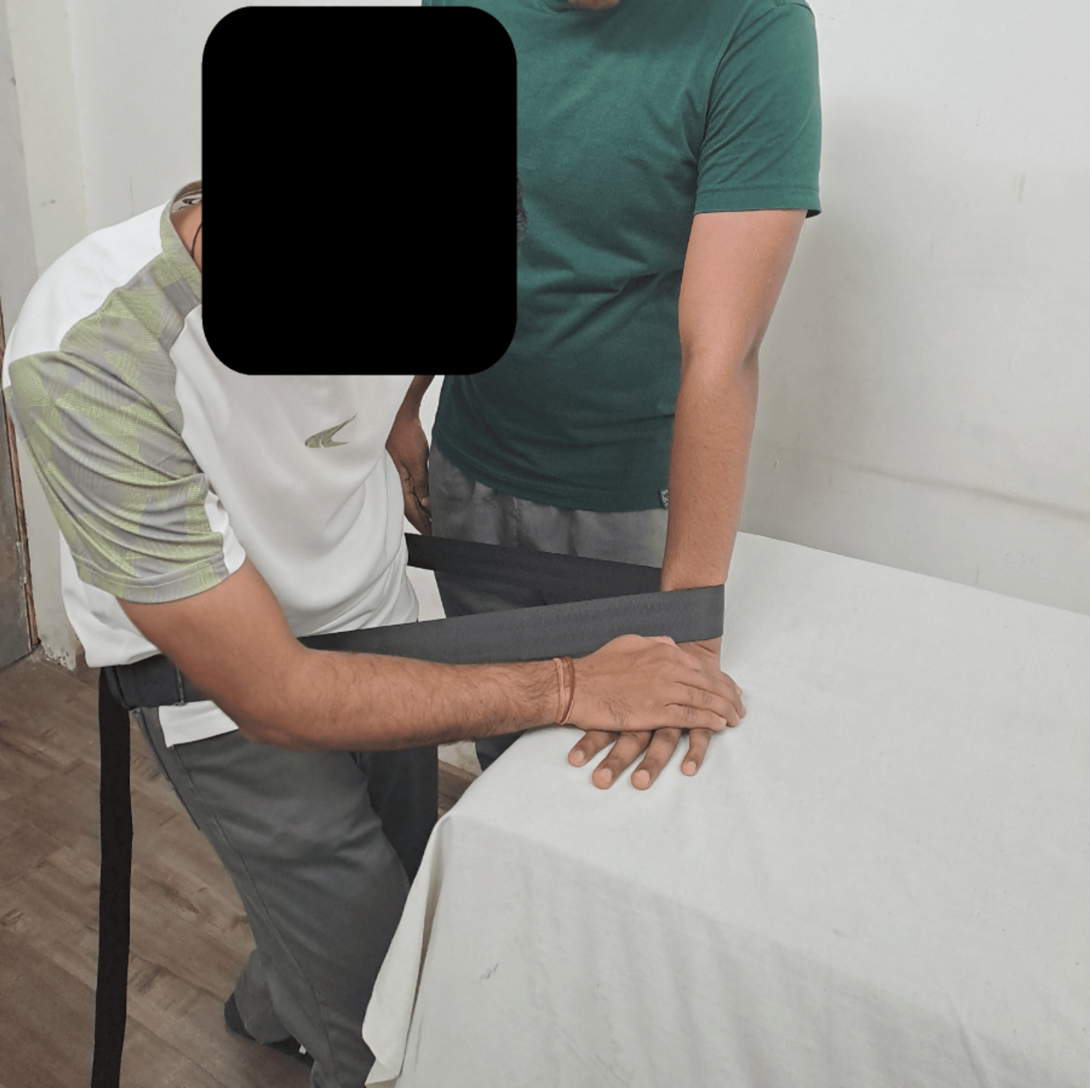
Therapist performing weight-bearing lateral (ulnar) glide for wrist joint with a Mulligan belt

Table 3 shows physiotherapeutic intervention [12-13]. Movement with mobilization is shown in Figure 3.

**Table 3:** Physiotherapeutic intervention

Goals	Intervention	Procedure	Rationale	Dosage
To relieve pain	Phonophoresis	Move the ultrasound head in circular motions over the localized pain area for five to 10 minutes	Utilizes ultrasound to enhance the delivery of a 2-g dose of 0.33% dexamethasone cream to affected tissues	1.0 W/cm^2^ at a 3-MHz frequency for five minutes in pulsed mode. Three sessions/week [14]
To provide support and stability	Kinesio taping	Measure and cut tape to fit the wrist. Apply with appropriate tension to support the injured structure	Provides external support to the wrist, limiting painful movements and promoting proprioception	The tape was applied at the start of each session and was kept in place for 48 hours. Upon completion of this period, the tape was removed, permitting the skin to rest for 24 hours prior to the next application. Following this 24-hour resting phase, the tape was reapplied, thereby providing continuous support during the treatment duration
Tissue healing	Laser therapy	Position the laser probe over the affected area. Administer therapy for five to 10 minutes	Low-level laser therapy promotes cellular metabolism and accelerates tissue repair	Three sessions/week
To correct the dysfunction	Movement with mobilization	Lateral glide for wrist using Mulligan belt	To reduce pain and improve range of motion	Six repetitions, three sets daily
To improve muscle strength and enhance wrist stability	Isometric strengthening	Instruct patient to contract wrist muscles without moving joint. Hold for five to 10 seconds, relax	Allows strengthening without exacerbating pain, maintaining muscle function around the wrist	Two times daily, three sets of 10 repetitions
	Flexor carpi radialis isometrics	Instruct patient to resist wrist flexion with palm facing upwards. Hold for five to 10 seconds, relax	Targets specific wrist flexor muscles essential for grip strength and wrist stability	Three times daily, three sets of 10-15 repetitions
	Eccentric strengthening with flex bar	Slowly lower the flex bar with the wrist in a flexed position. Return to starting position	Focuses on eccentric loading to improve tendon and ligament strength, crucial for joint stability	Three times per week, two to three sets of eight to 12 repetitions
To improve proprioception and coordination	Proprioception and coordination by using a stress ball	Place hands on the unstable ball. Perform controlled movements in neutral, pronation, and supination	Improves proprioceptive feedback and wrist stability through controlled movements on unstable surfaces	Three times per week, 10-15 minutes/session
	Upper limb kinetic chain conditioning	Perform activities that involve upper limb movements while focusing on wrist stability	Integrates wrist function into larger upper limb movements, promoting coordination and strength	Three sessions per week, five to 10 minutes/session
	Weight-bearing on one hand with a wobble board	Place hand on wobble board. Maintain plank position while balancing. Increase the difficulty as tolerated	Challenges wrist stability and proprioception through controlled weight-bearing activities	Three times per week, five to 10 minutes/session
To improve dynamic stability	Early low-load plyometrics	Start with dropping and catching a tennis ball. Progress to more dynamic movements as tolerated	Initiates controlled plyometric exercises to enhance wrist stability and prepare for higher-level activities	Gradually increase intensity and duration based on tolerance and function
Return to sports	High load plyometrics - return to sport	Perform sport-specific drills and exercises focusing on wrist stability and function	Simulates sport-specific movements to ensure readiness for return to basketball activities	Progressively increase intensity and duration

Outcome measure and follow-up

Table 4 shows the outcome measures used in the study.

**Table 4:** Pre- and post-outcome measures NPRS: numerical pain rating scale; DASH: disability of the arm, shoulder, and hand

Sr. No.	Outcome measures	Pre-treatment	Post-treatment
1	NPRS on movement	7/10	0/10
	NPRS at rest	2/10	0/10
2	Quick-DASH	50.7%	0%

## Discussion

Ganglion cysts, the most common benign soft tissue tumors of the hand, are often present on the dorsal wrist and are typically linked to synovial fluid herniation due to repetitive microtrauma or degeneration of the joint capsule [15-17]. The scapholunate ligament is a primary stabilizer of the wrist and is particularly susceptible to injury in individuals engaged in activities involving repetitive wrist motion or acute trauma [18-19]. Such injuries can lead to significant instability, characterized by pain, decreased range of motion, and reduced grip strength, severely impacting daily activities and athletic performance, ganglion cysts arising from the scapholunate ligament, are thought to develop due to myxoid degeneration of the ligament tissue itself. This degeneration leads to the formation of a cystic structure that can exacerbate the instability of the ligament, contributing further to the clinical symptoms [20-21]. These cysts can be particularly problematic as they are often adherent to the underlying ligament, complicating both diagnosis and treatment.

Choung et al. discovered that the application of mobilization techniques resulted in notable short-term improvements in alleviating dorsal wrist pain and increasing both active and passive ranges of motion in wrist extension for patients experiencing dorsal wrist pain [22]. These results indicate that mobilization techniques may be effectively advised to enhance wrist extension active and passive ranges of motion while also reducing dorsal wrist pain, especially during weight-bearing tasks involving the hand [22]. Given that the surgical excision of a dorsal wrist ganglion cyst may result in scapholunate instability due to potential intraoperative damage to the scapholunate ligament, surgeons must take measures to reduce such risks and to assess for instability in the postoperative period vigilantly. The ambiguity surrounding whether this complication is a result of surgical intervention or a pre-existing condition highlights the importance of thorough preoperative discussions with patients [23]. This situation further accentuates the significance of physical therapy, which serves not only as a conservative strategy to avert the need for surgery potentially but also as an essential element of rehabilitation to address or alleviate complications should surgery be performed. By enhancing wrist functionality and alleviating pain, physical therapy can significantly contribute to improved patient outcomes, regardless of the surgical difficulties encountered.

Tse et al. illustrated that a thorough rehabilitation program significantly alleviated pain and improved wrist functionality in individuals suffering from peri-scapholunate ligament injuries [24]. The initial stage of immobilization aims to minimize pain and promote soft-tissue recovery, which is succeeded by proprioceptive rehabilitation designed to enable pain-free engagement in daily activities and enhance strength [24]. Dias et al., in their case study, managed the ganglionic cyst with physical therapy with pain management, range of motion exercises, muscle strengthening, and training in activities of daily living [25].

The patient exhibited symptoms of pain, limited range of motion, and reduced grip strength, with MRI results confirming instability of the scapholunate ligament and the presence of a ganglion cyst. A customized rehabilitation protocol was developed to specifically address these concerns: phonophoresis and kinesio taping were implemented to alleviate pain and provide support, thereby minimizing stress on the ligament. Laser therapy facilitated tissue healing and decreased inflammation, supporting the recovery process. Strengthening exercises were incorporated to enhance muscle support and stability around the wrist. Each intervention was meticulously chosen to directly address the identified impairments, leading to significant enhancements in pain management, range of motion, and grip strength.

The patient's significant improvements in range of motion, muscle strength, functional performance, and reduced pain levels highlight the effectiveness of a well-structured physiotherapy regimen in managing SLL instability and associated ganglion cysts conservatively. This case underscores the importance of comprehensive physiotherapy in improving functional outcomes and facilitating a safe return to athletic activities, warranting further research and case studies to refine these conservative management strategies for similar musculoskeletal conditions.

## Conclusions

This case report underscores the effectiveness of conservative physiotherapy rehabilitation in managing scapholunate ligament instability accompanied by a ganglion cyst in a recreational basketball player. Through a comprehensive rehabilitation protocol targeting pain relief, stability, muscle strengthening, proprioception, and functional performance, significant improvements were observed in the patient's range of motion, muscle strength, and functional capabilities, with a substantial reduction in pain levels. The successful outcomes in this case highlight the potential of non-surgical interventions to restore wrist function and facilitate a safe return to athletic activities.
